# Obesity and cancer, a case for insulin signaling

**DOI:** 10.1038/cddis.2015.381

**Published:** 2015-12-31

**Authors:** Y Poloz, V Stambolic

**Affiliations:** 1 Division of Signaling Biology, Princess Margaret Cancer Centre/University Health Network, Toronto, Ontario, Canada; 2 Department of Medical Biophysics, University of Toronto, Toronto, Ontario, Canada

## Abstract

Obesity is a worldwide epidemic, with the number of overweight and obese individuals climbing from just over 500 million in 2008 to 1.9 billion in 2014. Type 2 diabetes (T2D), cardiovascular disease and non-alcoholic fatty liver disease have long been associated with the obese state, whereas cancer is quickly emerging as another pathological consequence of this disease. Globally, at least 2.8 million people die each year from being overweight or obese. It is estimated that by 2020 being overweight or obese will surpass the health burden of tobacco consumption. Increase in the body mass index (BMI) in overweight (BMI>25 kg/m^2^) and obese (BMI>30 kg/m^2^) individuals is a result of adipose tissue (AT) expansion, which can lead to fat comprising >50% of the body weight in the morbidly obese. Extensive research over the last several years has painted a very complex picture of AT biology. One clear link between AT expansion and etiology of diseases like T2D and cancer is the development of insulin resistance (IR) and hyperinsulinemia. This review focuses on defining the link between obesity, IR and cancer.

## Facts

- Hyperinsulinemia, along with the other obesity-related factors, is linked to the development of several types of cancers.
- Insulin, signaling through insulin receptor A, has direct oncogenic effects on cancer cells.
- Insulin-lowering drugs, such as metformin, may proof to be useful in lowering insulin levels and insulin resistance, decreasing body weight and improving cancer outcomes in patients with obesity and type 2 diabetes.

## Open Questions

- How are the PI3K-AKT and the Ras-MAPK pathways regulated by INSR-A in normal epithelial cells and in cancer?
- How should obesity and T2D be treated in order to minimize the risk of cancer development, specifically keeping in mind the potential oncogenic effect of hyperinsulinemia?
- What are the effective drugs targeting the molecular pathways that link obesity and T2D to cancer?

## Obesity and Cancer

**Figure 1:**
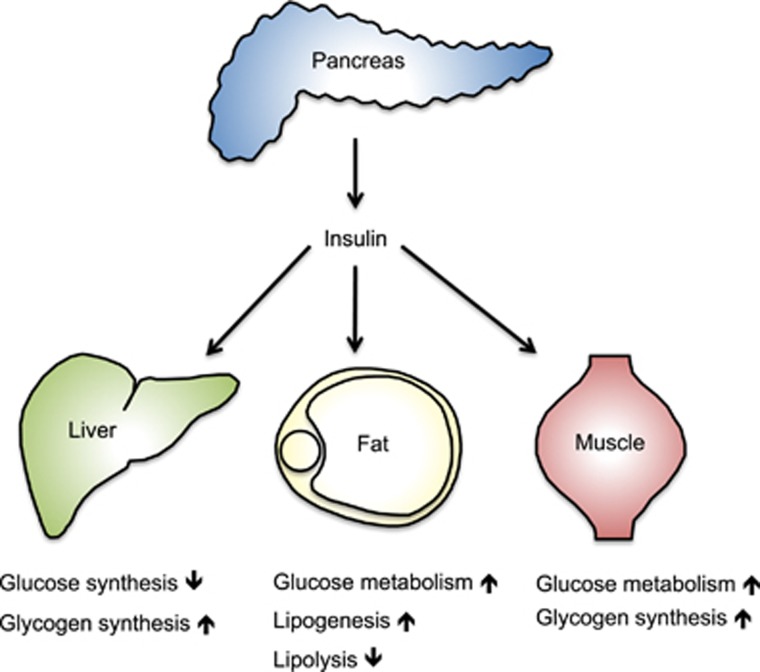
The role of insulin in the control of whole-body metabolism

Insulin is the master regulator of energy storage and whole-body metabolism (Figure 1). It is produced and secreted by pancreatic *β* cells in response to a surge in blood glucose levels. Insulin stimulates glucose uptake by adipose tissue (AT) and muscle, whereas suppressing the release of glucose from the liver. It also stimulates the liver and the muscle to store excess glucose in the form of glycogen. In addition to regulating glucose homeostasis, insulin also induces fat storage. In adipocytes, it inhibits lipolysis while inducing lipogenesis and fatty acid uptake from the blood stream. Insulin thus ensures sufficient storage of energy that can be mobilized during fasting, when insulin levels are low.

Perpetual caloric excess in individuals with obesity disrupts the intricate balance between energy storage and mobilization, leading to desensitization of tissues to the actions of insulin and the development of IR. The attenuation of the response of insulin target tissues to the physiological actions of insulin leads to a compensatory increase in pancreatic insulin production in an attempt to reestablish glucose homeostasis and thus overt hyperglycemia. This overproduction of insulin by the pancreatic *β* cells and a concomitant increase in serum insulin levels is a condition called hyperinsulinemia.

Strong clinical and epidemiological evidence links hyperinsulinemia, along with the other obesity-related factors, to the development of several types of cancers, including those of the breast, endometrium, colon, liver, esophagus, kidney and pancreas.^1, 2, 3, 4, 5, 6, 7^ International Agency for Research on Cancer estimated that obesity is a cause of 11% of colon, 9% of postmenopausal breast, 39% of endometrial, 25% of kidney and 37% of esophageal cancer cases.^7^ A prospective study of 900 000 adults in the United States reported that obesity accounts for 14% of deaths from cancer in men and 20% in women, directly linking excess body weight to cancer mortality.^3^ This study also highlighted a dose–response relationship between obesity and cancer, finding an increase in cancer risk with increasing body mass index (BMI). Finally, an analysis of the global burden of cancer has identified that 3.6% of all new cancers are attributable to BMI.^8^ Of particular interest, weight loss following a lifestyle change or bariatric surgery reduces cancer risk,^9, 10, 11^ whereas in the Swedish Obesity Subjects study, women who lost >30% of their body weight had a marked 41% reduction in cancer risk.^11^ Although some recent research suggests that the hereditary and environmental factors contribute less to the cell transformation than the cell's division history,^12^ it is likely that the interplay of the cumulative changes associated with cell's proliferative past, in the context of the genetic underpinning and the environmental factors such as obesity, underlies the development of most cancers. The beneficial effects of weight loss raise the possibility that the adverse impact of obesity on cancer can be reversed and suggests that obesity-directed therapies may impact cancer treatment and survival.

## The Insulin Signaling Pathway

**Figure 2:**
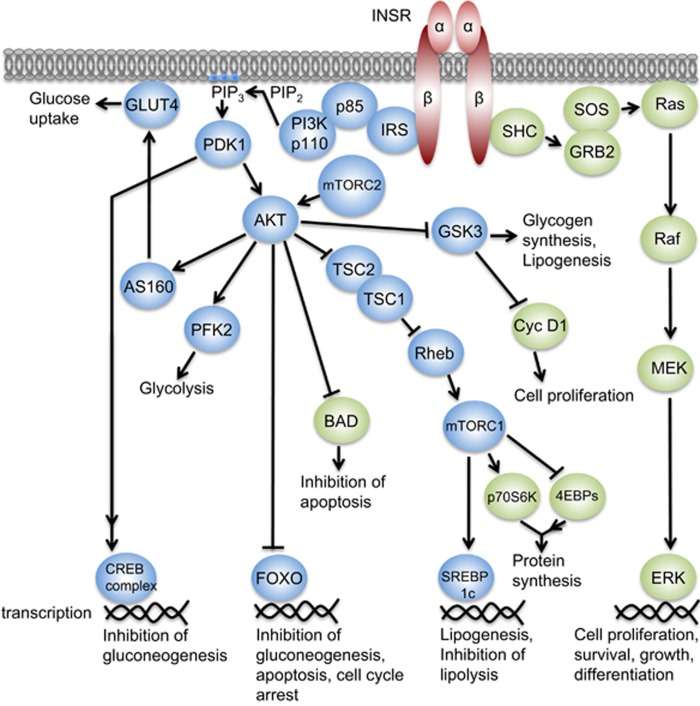
The insulin signaling pathway

**Figure 3:**
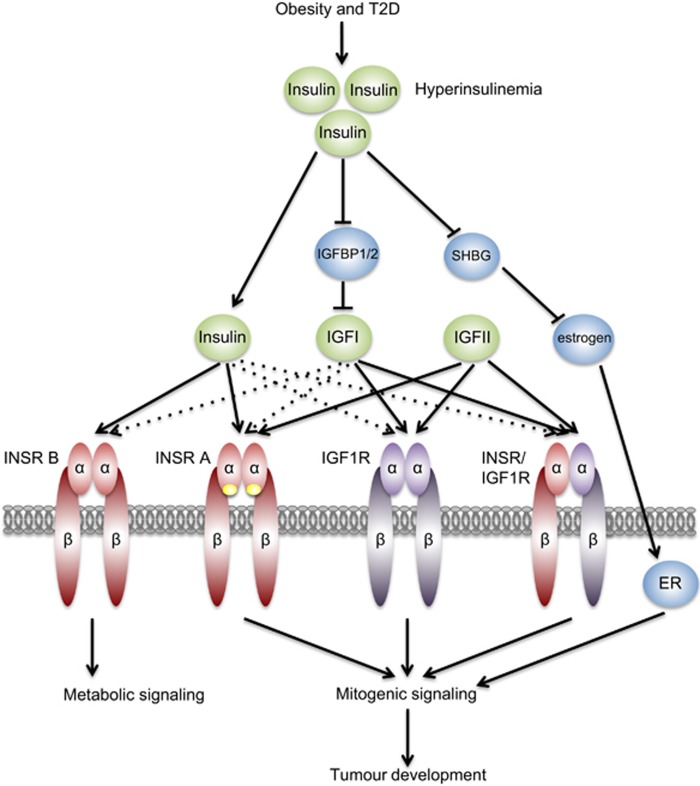
The link between hyperinsulinemia and cancer development

The diverse cellular actions of insulin are initiated by its binding to the insulin receptor (INSR) on the surface of target cells (Figure 2). The INSR signaling pathway mediates both the metabolic and the mitogenic effects of insulin and its deregulation is central to the development of insulin resistance (IR). INSR is a heterotetramer composed of two extracellular insulin-binding *α* subunits (130 kDa), covalently linked to two transmembrane *β* subunits (95 kDa) with intrinsic tyrosine kinase activity.^13^ There are two isoforms of INSR, INSR-A and INSR-B, differing by the presence (INSR-B) or the absence (INSR-A) of exon 11, which encodes a 12 amino-acid stretch at the C-terminal end of the *α* subunit (Figure 3).^14, 15, 16^ The lack of exon 11 in INSR-A allows this receptor to bind not only insulin but also insulin-like growth factor II (IGFII) and pro-insulin with high affinity.^17, 18^ Indeed, INSR-A has a 1.7-fold higher affinity for insulin than INSR-B and is internalized and recycled faster than INSR-B.^19, 20, 21^

The expression of the two isoforms is regulated developmentally and in a tissue-specific manner. The INSR-A isoform is predominantly expressed in the fetal tissues where it regulates embryonic growth. Remarkably, INSR-A is also the predominant isoform overexpressed by many cancer cells.^22, 23, 24^ In human embryos, lack of insulin, as in the cases of pancreatic agenesis, lack of *β* cells or pancreatic islets, or transient neonatal diabetes, results in severe growth retardation.^25, 26, 27^ INSR deficiency during embryonic development results in comparable growth retardation, as seen in the infants with the Donohue syndrome.^28^ INSR-A also mediates the mitogenic signaling in the regulation of pancreatic *β*-cell proliferation, and is able to protect the myeloid 32D cells from apoptosis upon IL-3 removal more effectively than INSR-B.^23, 29^ INSR-B is predominantly expressed in the differentiated adult tissues, particularly the liver, the fat and the muscle, where it regulates the metabolic effects of insulin. An abnormally high INSR-A:INSR-B ratio in muscle cells of patients with myotonic dystrophy appears to be responsible for the IR seen in these patients, further highlighting the functional differences between the two isoforms of the receptor.^30^ In mice, INSR deletion leads to 10–20% growth retardation and metabolic abnormalities that develop after birth.^31^

INSR can form hybrid receptors from INSR-A or B but also with a related tyrosine kinase, insulin-like growth factor 1 receptor (IGF1R), thus resulting in a complex, tissue-specific regulation of the metabolic and the mitogenic signaling pathways. Upon insulin binding, INSR undergoes a conformational change and autophosphorylates several residues in the C-terminal tail of the *β* subunit, leading to recruitment and further phosphorylation of a number of effector proteins. Insulin receptor substrates (IRSs), which bind INSR and are directly phosphorylated by it on tyrosine residues, present sites for binding of adaptor molecules containing Src homology 2 (SH2) and phosphotyrosine-binding (PTB) domains, which further propagate the signals.^32, 33^ One such effector is the regulatory subunit (p85) of phosphoinositide 3-kinase (PI3K), which, when bound to tyrosine phosphorylated IRS, relieves inhibition of the catalytic PI3K subunit (p110) leading to its activation.^34^ PI3K then phosphorylates phosphatidylinositol 4,5-bisphosphate (PIP2) to produce phosphatidylinositol 3,4,5-triphosphate (PIP3), a cell membrane-associated lipid second messenger that attracts proteins containing pleckstrin homology (PH) domains, including protein kinase B/AKT (PKB/AKT) and the 3-phosphoinositide-dependent protein kinase 1 (PDK1).^35^ PIP3 recruits AKT and PDK1 to the plasma membrane where PDK1 phosphorylates AKT on threonine 308 (T^308^), leading to its partial activation.^36^ Mammalian target of rapamycin complex 2 (mTORC2) then phosphorylates AKT on serine 473 (S^473^), fully activating the protein.^37^

AKT signaling through numerous downstream targets governs the metabolic effects of insulin. For example, the AKT substrate of 160 kDa (AS160) regulates the glucose transporter 4 (GLUT4) translocation to the plasma membrane, a process that initiates cellular glucose uptake in response to insulin.^38^ Phosphorylation of the phosphofructokinase 2 by AKT induces glycolysis, enabling cells to metabolize glucose into usable energy in the form of adenosine triphosphate (ATP).^39^ AKT also phosphorylates and inhibits the glycogen synthase kinase 3 (GSK3), a negative regulator of glycogen synthesis and lipogenesis.^40, 41^ In the liver, PDK1-dependent disassembly of the cAMP-response-element-binding protein (CREB) complex and phosphorylation of the forkhead box O (FOXO) by AKT suppress gluconeogenesis.^42, 43, 44^ Finally, AKT phosphorylates and downregulates the GTPase activator protein (GAP) activity of tuberous sclerosis complex 2 (TSC2) toward Ras homolog enriched in brain (Rheb), a G protein that regulates the activity of mammalian target of rapamycin complex 1 (mTORC1).^45, 46, 47, 48^ This allows for a Rheb-mediated activation of mTORC1, which in turn phosphorylates the sterol regulatory element-binding protein 1c (SREBP1c), a transcription factor that induces the transcription of genes involved in lipogenesis and represses those involved in lipolysis.^49, 50^

The PI3K-AKT pathway also mediates some of the mitogenic effects of insulin. mTORC1 regulates cell growth not only through SREBP1-mediated lipid synthesis but also by controlling mRNA translation, via direct phosphorylation of the S6 kinase (p70S6K) and the 4E binding proteins (4EBPs).^51, 52^ In addition, AKT-mediated phosphorylation and deactivation of the transcription factor FOXO results in its nuclear export and proteasomal degradation, thus releasing cells from FOXO-mediated cell cycle arrest.^53, 54, 55^ Deactivation of FOXO, along with another target of Akt activity, the BCL2-associated agonist of cell death (BAD), coordinately represses the cellular apoptotic program.^56^ Finally, AKT-mediated inhibition of GSK3 results in nuclear accumulation of cyclin D1, which regulates G1/S phase transition.^57^

The mitogenic effects of insulin are mainly mediated by the recruitment of SH2 domain-containing adaptor (SHC) to INSR and activation of the rat sarcoma-mitogen-activated protein kinase/ERK (Ras-MAPK/ERK) signaling pathway.^58^ SHC interacts with the activated INSR via its PTB domain and recruits growth factor receptor bound 2 – son of sevenless (GRB2-SOS) complexes to the cell membrane where SOS acts as a guanine nucleotide exchange factor (GEF) for Ras, converting GDP-bound Ras into the active GTP-bound form.^59, 60, 61, 62^ Ras may also be activated through the interaction of the GRB2-SOS complex with IRSs.^62^ Ras then recruits and activates Raf, which phosphorylates and activates MEK, which in turn phosphorylates and activates MAPK/ERK.^63, 64^ Activated MAPK translocates to the nucleus where it phosphorylates several transcription factors that regulate genes involved in cell growth, proliferation, differentiation and survival.^65^ Activated Ras can also feed into the PI3K-AKT signaling pathway through its direct interaction with PI3K p110, independently of p85.^66^ Multiple negative feedback loops and crosstalk between the PI3K-AKT and RAS-MAPK pathways orchestrate the dynamic and intricate tissue-specific effects of insulin, governing both cell metabolism and proliferation.

How the two isoforms of INSR differentially activate the mitogenic *versus* the metabolic signaling pathways in cells is under active investigation, especially as many cancer cells overexpress INSR-A. In the pancreatic *β* cells, insulin upregulates the expression of its own gene through INSR-A and the IRS2-PI3K class Ia-mTORC1-p70S6K signaling pathway,^67, 68^ whereas the upregulation of the glucokinase (*β*GK) gene requires the INSR-B signaling through the PI3K class II C2*α*-PDK1-PKB/AKT signaling pathway.^69^ Work in MEFs engineered to only express either INSR-A or INSR-B (R^-^ cells), revealed that long-acting insulin analogs (administered to many patients with diabetes) preferentially stimulated cell proliferation and led to higher ERK:AKT phosphorylation ratios through INSR-A.^70^ Differential activation of the signaling pathways by the two INSR isoforms can, at least in part, be attributed to their distinct localization within the plasma membrane and discrete internalization and recycling dynamics.^21, 71^

## Insulin Signaling and Cancer

INSR is often overexpressed in tumor cells, particularly that of the breast.^72, 73, 74, 75, 76^ Increased INSR expression in breast tumors is associated with poor survival in patients.^74^ INSR is also overexpressed and highly phosphorylated in mammary tumors from diabetic mice.^77^ Preferential expression of the INSR-A isoform has been demonstrated in cancers of the breast, lung, colon, ovaries, endometrium, thyroid and muscle.^18, 73, 78, 79, 80, 81, 82^ *In vitro*, INSR-A has been shown to be essential for the growth and survival of many cancer cell lines, including those of the breast.^82, 83, 84, 85^ Overexpression of INSR-A in NIH 3T3 fibroblasts or in immortalized human breast epithelial cells, induced a ligand-dependent transformed phenotype, which could be reversed with anti-INSR antibodies.^86, 87^ INSR is also essential for virus-induced transformation of vascular endothelial cells.^88^ Finally, the knockdown of INSR in MDA-MB-435 breast cancer cell line results in smaller xenograft tumors and fewer pulmonary metastases.^89^

A pathway initiated by the IGFIR receptor operates in parallel to the INSR, sharing several downstream components. This pathway can be activated by three ligands: insulin, IGFI and IGFII (Figure 3). Hyperinsulinemia has been shown to increase hepatic production and bioavailability of IGFI, in part by inhibiting hepatic production of IGF binding proteins 1 and 2 (IGFBP1 and IGFBP2), which sequester IGFs in the serum.^90, 91^ This excess IGFI may hyperactivate IGF1R and INSR/IGFIR and their proliferative and anti-apoptotic programs in both premalignant and malignant tissues. Strong evidence for a direct and independent impact of hyperinsulinemia on cancer development came out of studies in the MKR mice.^77^ These animals express a dominant negative form of IGF1R in the muscle, thus disrupting signaling through the IGF1R receptors and INSR/IGF1R hybrid receptors. This results in severe muscle IR and systemic hyperinsulinemia, without obesity, hyperglycemia or hyperlipidemia, thus allowing an assessment of the impact of high insulin levels on cancer, without the confounding effects of other obesity-related factors. When implanted with xenografts, MKR mice formed larger mammary tumors, with increased lung metastases compared with their non-hyperinsulinemic wild-type counterparts. Chronic treatment of MKR mice with a fast-acting insulin analog (AspB10) results in even bigger tumors and a further increase in INSR but not IGF1R phosphorylation. In this model, tumors display greater INSR but not IGF1R phosphorylation.^92, 93^ Accordingly, knockdown of INSR, but not IGF1R, in mammary carcinoma Mvt-1 cell line led to the considerably smaller xenografted tumors, both in wild-type and hypersinsulinemic mice.^94^ Similarly, in breast cancer patients, increased INSR but not IGFIR expression and higher phosphorylation of INSR/IGFIR hybrid receptors correlate with poor survival.^74^

In addition to the possible direct oncogenic effects of insulin on the proliferative and anti-apoptotic signaling in cancer cells, insulin is also implicated in various aspects of the maintenance of whole-body homeostasis, including the action of sex hormones, which may contribute to carcinogenesis. For over 100 years, sex hormones have been known to affect cancer development and progression.^95^ Hyperinsulinemia inhibits hepatic production and secretion of the sex hormone-binding globulin (SHBG; Figure 3).^96, 97^ A reduction in serum SHBG results in increased bioavailability of estradiol (in men and in women) and testosterone (in women only).^98, 99^ High insulin levels can also increase ovarian and adrenal androgen production.^100^ Moreover, adipocytes express a number of sex steroid stabilizing enzymes that convert androgenic precursors into estrogens, thus AT can act as a sink or a source of lipid-soluble sex hormones.^101^ Remarkably, AT is the main site of estrogen production in men and postmenopausal women.^102, 103^ Considering that >70% of all breast cancers express the estrogen receptor (ER) and that activation of this receptor initiates a proliferative and anti-apoptotic transcription program, in such tumors, deregulated insulin signaling and its effects on AT may have considerable impact on prognosis and outcome.^99, 104^

## Hyperinsulinemia Drugs as Cancer Therapeutics

Metformin is the most commonly used drug for treatment of type 2 diabetes (T2D). It is cheap, widely available and well tolerated. Growing preclinical, clinical and epidemiologic evidence suggests that metformin may also prove to be a valuable drug for cancer therapy. Patients with diabetes that are treated with metformin have a lower incidence and mortality of breast, pancreatic, hepatocellular and colorectal cancer.^105, 106, 107, 108, 109, 110^ Metformin may impact tumor cells via direct and indirect mechanisms, both involving the activation of 5′-AMP-activated protein kinase (AMPK), the major cellular sensor of energy stress. Metformin is transported into cells via the organic cation transporter 1 (OCT1), where it accumulates in the mitochondria and inhibits the complex 1 of the mitochondrial respiratory chain.^111, 112^ Inhibition of mitochondrial ATP synthesis by metformin leads to a rise in intracellular AMP, which binds AMPK and facilitates its phosphorylation by the liver kinase B1 (LKB1) on threonine 172, thus activating the protein.^113, 114^ In the liver, AMPK-mediated suppression of gluconeogenesis results in a decrease in the levels of fasting blood glucose, leading to a reduction in circulating insulin levels and resensitization of insulin target tissues to the action of insulin.^115, 116^

A decrease in circulating insulin levels may also result in the downregulation of INSR signaling pathways in cancers expressing INSR and attenuation of the proliferative and anti-apoptotic signals^117, 118^ Supporting such a possibility, non-diabetic breast cancer patients given metformin for 6 months displayed an average 22% reduction in insulin levels.^119^ Moreover, a 2-week administration of metformin in between diagnosis and surgery led to a reduction in circulating insulin levels, a decrease in INSR expression and downregulation of AKT and MAPK signaling pathways in their tumors.^117^

Metformin may also have direct effects on tumor cells, involving AMPK-mediated stabilization of TSC2 and concomitant inhibition of mTORC1. This results in the downregulation of p70S6K and 4EBP1 activities and inhibition of protein synthesis and cell proliferation.^120, 121, 122, 123^ Limited evidence also suggests that there may be other, AMPK independent, direct effects of metformin on cancer cells.^124^ *In vitro* data on metformin action should be interpreted with caution though, as the concentrations of the drug used in such studies far exceed tolerable doses achievable in humans.^125^

To further probe the utility of metformin in cancer therapy, the NCIC Clinical Trials Group, as part of the North American Breast Cancer Group, is leading an ongoing phase III, randomized and placebo-controlled clinical trial (MA.32).^115^ The effect of metformin on disease-free survival and other outcomes is monitored in 3649 non-diabetic women with early-stage breast cancer receiving metformin *versus* placebo for 5 years. Analyses of key metabolic parameters after 6 months of treatment showed that women on metformin have a reduction in body weight (−3.0%), glucose (−3.8%), insulin (−11.1%), IR (homeostasis model assessment; −17.1%) and leptin (−20.2%). Furthermore, there was a significant reduction in the levels of highly sensitive C-reactive protein (−6.7%), a marker of chronic inflammation. Further analyses in this cohort are aimed at identifying the link(s) between metformin's metabolic and anti-inflammatory impact on cancer-free survival.

## Sources of IR in Obesity

**Figure 4:**
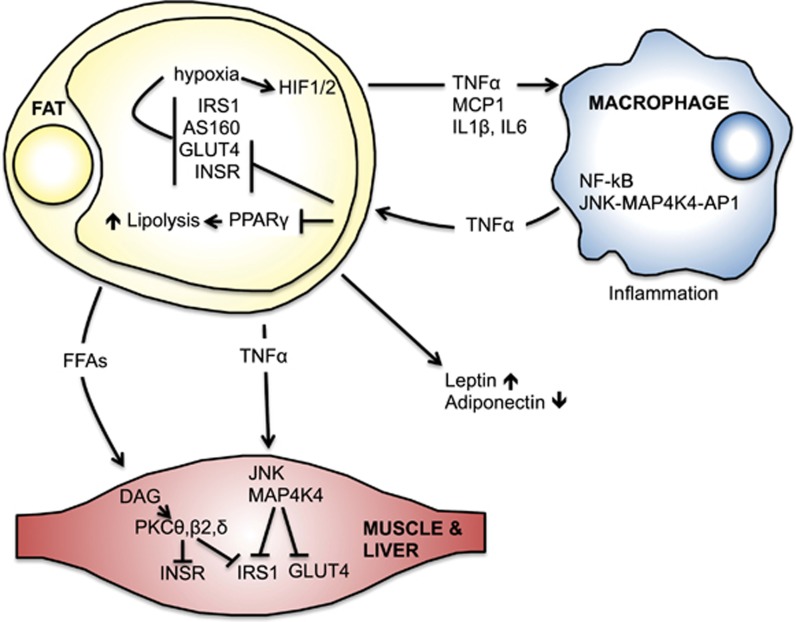
The sources of IR in obesity

A series of confounding factors and a complex interplay between many tissues contributes to the development of IR and hyperinsulinemia in obesity. The mechanisms have not been fully elucidated, but the major factors center on the changes in AT biology, disruption of the normal endocrine function of adipocytes, as well as deregulated lipolysis and overproduction of free fatty acids (FFAs; Figure 4).

AT is the body's main energy reserve. There are two major types of AT, white AT (WAT) and brown AT (BAT). BAT is primarily responsible for heat production in infants, although small BAT depots have recently also been identified in adults.^126^ The majority of body fat is stored in WAT, where adipocytes store energy in the form of triglycerides (TAGs). When there is energetic demand, TAGs are broken down into glycerol and FFAs through the process of lipolysis. Adipocytes then release glycerol and FFAs into the blood stream for transporting to target tissues, mainly the liver and the muscle. Oxidative metabolism in these tissues breaks down the FFAs to produce ATP, whereas gluconeogenesis in the liver converts glycerol into glucose.

Fat storage is not the only function of AT, as adipocytes are also secretory cells. They produce a number of hormones and cytokines, now termed adipokines, which influence AT biology, as well as cellular metabolism and function in the brain, liver, muscle, vasculature, reproductive organs and *β* cells of the pancreas.^127^ This function of adipocytes came to light when *leptin* was discovered as the gene mutated in obese *ob/ob* mice.^128^ Leptin is a peptide hormone, produced primarily by the adipocytes, which opposes many actions of insulin. It acts in the hypothalamus to repress appetite, and in the liver and AT to stimulate lipolysis and inhibit lipogenesis through activation of AMPK.^129, 130^ When administered to *ob/ob* mice or human patients with a mutation in the *leptin* gene, leptin lowers blood glucose levels and resensitizes cells to insulin.^131, 132^ Obesity in humans often leads to high leptin levels and leptin resistance.^133, 134^ Adiponectin is another hormone product of adipocytes.^135^ In the muscle and liver, adiponectin binding to its receptor activates two signaling pathways, involving LKB1 and the Ca^2+^/calmodulin-dependent protein kinase kinase (CaMKK), which converge on AMPK activation, which then induces glucose uptake and lipolysis, inhibits gluconeogenesis and promotes FFA oxidation.^136, 137^ Adiponectin also sensitizes cells to the action of insulin by inducing transcription of IRS and GLUT4, as well as translocation of GLUT4 to the membrane.^138, 139, 140, 141^ Paradoxically, adiponectin levels are low in patients with obesity and/or T2D, which likely contributes to the development of IR.^142, 143^ A perturbed balance in leptin and adiponectin levels is a hallmark of obesity and IR.

Adipocytes also produce pro-inflammatory cytokines, such as the tumor necrosis factor alpha (TNF*α*), monocyte chemoattractant protein 1 (MCP-1), interleukins 1*β* and 6 (IL1*β* and IL6) and others.^144, 145, 146^ TNF*α*, like leptin, stimulates lipolysis and inhibits lipogenesis in AT.^147^ Enlarged adipocytes from the obese rodents and humans overexpress TNF*α*, which is thought to have a major role in the development of IR.^148, 149^ In the muscle, TNF*α* leads to activation of the Jun N-terminal kinase 1 (JNK1) and mitogen-activated protein kinase kinase kinase kinase 4 (MAP4K4) pathway, which inhibits GLUT4 translocation to the membrane and induces inhibitory serine (S^270^, S^307^ and S^636/639^) phosphorylation of IRSs, thereby inhibiting INSR signaling.^150, 151, 152, 153, 154, 155^ In adipocytes, TNF*α* inhibits IRS and GLUT4 expression.^156^ Highlighting the physiological relevance of these relationships, knockout of TNF*α* or MCP-1 in high-fat diet-fed mice, or knockdown of MAP4K4 in the isolated muscles from patients with diabetes, ameliorates IR.^149, 150, 157^ TNF*α* also impedes expression and protein stability of the nuclear hormone receptor peroxisome proliferator-activated receptor gamma (PPAR*γ*).^158, 159^ This transcription factor regulates expression of genes involved in lipogenesis and lipid sequestration in adipocytes and thus a reduction in its expression leads to overproduction of FFAs. PPAR*γ*-activating thiazolidinediones or the activation of PPAR*γ* by deletion of NCoR, corepressors of transcription, can block sensing of cytokines and improve insulin sensitivity in adipocytes.^160, 161^

MCP-1, which is also overexpressed in AT during obesity, acts as a chemoattractant for macrophages and other immune cells that infiltrate AT, leading to the development of inflammation, another hallmark of obesity and IR.^146, 162, 163^ AT of individuals with obesity consists of up to 50% of macrophages, whereas they only make up 5–10% of AT cells in lean subjects.^164^ Macrophages locally secret TNF*α*, a process that requires signaling by the nuclear factor kappa-light-chain-enhancer of activated B cells (NF-kB) and JNK-MAP4K4-AP1 (activator protein 1) pathways.^165, 166, 167^ Indicative of the importance of TNF*α* in AT biology, knockout of JNK1 in macrophages partially protects from high-fat diet-induced IR in mice.^167^ Consistent with such a notion, anti-inflammatory drugs like the salsalate, an NF-kB inhibitor, have shown some efficacy in improving IR in patients with obesity and T2D.^168, 169, 170^

Rapid expansion of AT mass and enlarged adipocyte size in obesity impact oxygen delivery and can create hypoxic stress, with further effects on the development of IR. In adipocytes in culture, hypoxia leads to reduced INSR and IRS1 tyrosine phosphorylation, directly affecting their ability to sense insulin.^171^ Hypoxia has also been found to affect the glucose transport machinery via the reduction in AS160 phosphorylation and eventually lowered GLUT4 expression.^171^ Finally, low O_2_ pressure in adipocytes stabilizes the O_2_-sensitive transcription factor hypoxia-inducible factor 1 alpha (HIF1*α*), the master regulator of cellular response to hypoxia.^172^ HIF1*α*, in concert with HIF1*β*, induces expression of TNF*α*, IL6 and leptin, whereas repressing expression of adiponectin, further driving IR development.^173, 174^

Another major hallmark of AT expansion is an increase in circulating FFA levels, because of elevated lipolysis, and their uptake by the muscle, liver and pancreatic cells. Excessive FFA accumulation in these cells results in lipotoxicity and development of IR.^175^ There are several hypotheses as to how excess FFAs lead to IR. One suggests that an increase in diacyglycerol levels in the muscle, likely due to a backlog in the FFA reesterification pathway, leads to activation of protein kinase C theta, beta2 and delta (PKC*θ*,*β*2,*δ*).^176, 177, 178, 179^ These novel PKCs phosphorylate INSR and IRSs on inhibitory serine residues thereby reducing their activity. Another hypothesis suggests that FFAs induce the pro-inflammatory response through activation of NF-kB signaling and production of pro-inflammatory cytokines like TNF*α*, MCP-1, IL6 and IL1*β*.^180^ The development of IR in response to FFAs is thus a complex process, involving deactivation of INSR signaling pathway components and induction of inflammation and cellular stress.

Insufficiency in AT, as seen in the cases of lipodystrophy, can also lead to IR. As AT is a storage depot for fat, physiologically low AT levels lead to elevated circulating concentrations of TAG and FFAs and development of IR.^181, 182^ Healthy AT is also required for proper secretion and the physiological balance of adipokines like adopinectin and leptin, which sensitize cells to insulin.^183, 184^ Leptin replacement therapy is now an approved treatment for patients with lipodystrophy, and significantly attenuates IR in those patients.^185^ Functional AT in proportion to body size is thus essential for normal insulin sensitivity and whole-body metabolism.

## Outlook and Treatment Options

Special considerations have to be made for the prevention, detection and treatment of cancers in patients with obesity and T2D. In 2007, the World Cancer Research Fund/American Institute for Cancer Research Second Expert Report provided the guidelines for cancer prevention, with the main recommendation being ‘maintaining a healthy weight throughout life'.^6^ Thus, patients should be encouraged to lose weight and undergo a lifestyle change, incorporating exercise and healthy diet into their daily lives.

There are several challenges in cancer detection in the overweight/obese patients. Women with obesity and T2D are less likely to use the preventative services for cancer screening.^186, 187^ Among over 700 000 Canadian women, those with diabetes were 32% less likely to receive routine mammography screening.^186^ This highlights a need for better patient education and organization of primary healthcare to ensure early cancer detection in this high-risk group. Obesity is also linked to a reduction in tumor biomarkers, like the carcinoembryonic antigen in colorectal cancer.^188^ The ability to detect cancer in individuals with obesity is further complicated by the restrictions in the weight and the diameter of most imaging modalities, as well as poorer quality of the collected images.^189^ A considerable challenge in the treatment of cancer in these patients is the ability to achieve proper chemotherapy dosing. In a study of over 9000 women with breast cancer, reduced chemotherapy dosing was found in up to 40% of patients with obesity.^190^ Furthermore, patients with T2D and obesity may present for cancer treatment with pre-existing renal, cardiovascular or neurological complications, conditions that can be further exacerbated by the chemotherapeutic agents. The use of glucocorticoids should be carefully considered, as they are known to cause IR, decrease insulin production and secretion, as well as increase gluconeogenesis and glycogenolysis.^191^ Inflammation has long been associated with neoplastic transformation and the inflammatory cytokines are known to activate oncogenic signaling in cells. Thus, targeting the obesity-associated inflammation may reduce the development of IR while decreasing the oncogenic input from the cytokines in the tumor microenvironment, together inhibiting cancer development.^192^

The ability to treat cancers in patients with obesity and T2D will hinge on the development of effective drugs targeting the molecular pathways that link obesity and T2D to cancer. For example, targeting INSR-A or the PI3K-AKT and/or RAS-MAPK pathways that are downstream of INSR-A represents such an option. Moreover, supplementing the standard of care treatments with metformin or other insulin-lowering drugs may proof to be useful in lowering insulin levels and IR, decreasing body weight and improving cancer outcomes in patients with obesity and T2D.

## Abbreviations

T2D: type 2 diabetes
BMI: body mass index
AT: adipose tissue
IR: insulin resistance
INSR: insulin receptor
INSR-B: insulin receptor B isoform
INSR-A: insulin receptor A isoform
IGFII: insulin-like growth factor II
IGF1R: insulin-like growth factor receptor 1
IRSs: insulin receptor substrates
SH2: Src homology 2
PTB: phosphotyrosine-binding
p85: p85 regulatory subunit of PI3K
PI3K: phosphoinositide 3-kinase
p110: p110 catalytic subunit of PI3K
PIP2: phosphatidylinositol 4,5-bisphosphate
PIP3: phosphatidylinositol 3,4,5-triphosphate
PH: pleckstrin homology
PKB/AKT: protein kinase B
PDK1: 3-phosphoinositide-dependent protein kinase 1
mTORC2: mammalian target of rapamycin complex 2
AS160: AKT substrate of 160 kDa
GLUT4: glucose transporter 4
GSK3: glycogen synthase kinase 3
CREB: cAMP-response-element-binding
FOXO: forkhead box O
GAP: GTPase activator protein
TSC2: tuberous sclerosis complex 2
Rheb: Ras homolog enriched in brain
mTOCR1: mammalian target of rapamycin complex 1
SREBP1c: sterol regulatory element-binding protein 1c
p70S6K: S6 kinase
4EBPs: 4E binding protein
BAD: BCL2-associated agonist of cell death
SHC: Src homology 2 domain-containing
Ras: rat sarcoma
MAPK/ERK: mitogen-activated protein kinase
GRB2: growth factor receptor bound 2
SOS: son of sevenless
GEF: guanine nucleotide exchange factor
GK: glucokinase
IGFBP: IGF binding protein
AspB10: fast-acting insulin analog
SHBG: sex hormone-binding globulin
ER: estrogen receptor
AMPK: 5′-AMP-activated protein kinase
OCT1: organic cation transporter 1
LKB1: liver kinase B1
FFAs: free fatty acids
WAT: white adipose tissue
BAT: brown adipose tissue
TAGs: triglycerides
ATP: adenosine triphosphate
CaMKK: Ca^2+^/calmodulin-dependent protein kinase kinase
MCP-1: monocyte chemoattractant protein 1
IL: interleukin
JNK1: Jun N-terminal kinase
MAP4K4: mitogen-activated protein kinase kinase kinase kinase 4
PPAR*γ*: proliferator-activated receptor gamma
NF-kB: nuclear factor kappa-light-chain-enhancer of activated B cells
AP1: activator protein 1
HIF1*α*: hypoxia-inducible factor 1 alpha
PKC*θ*: protein kinase C theta
PKC*β*2: PKC beta 2
PKC*δ*: PKC delta.
